# Functional, Radiographic, and Patient-Reported Outcomes of Hybrid Fixation Versus Posterior Long Fusion for Double-Major Adolescent Idiopathic Scoliosis

**DOI:** 10.3390/jcm15166340

**Published:** 2026-08-17

**Authors:** Burak Abay, Selmin Ervin Arsoy, Hamisi Mwarindano Mraja, Ilyas Dolas, Meric Enercan, Azmi Hamzaoglu

**Affiliations:** 1 Department of Orthopedics and Traumatology, Demiroglu Bilim University, 34381 Istanbul, Turkey; 2 Department of Physiotherapy and Rehabilitation, Demiroglu Bilim University, 34381 Istanbul, Turkey; 3 Scoliosis and Spine Center—Istanbul, Istanbul Florence Nightingale Hospital, 34381 Istanbul, Turkey; 4 Department of Neurosurgery, Demiroglu Bilim University, 34381 Istanbul, Turkey

**Keywords:** adolescent idiopathic scoliosis, hybrid fixation, vertebral body tethering, posterior spinal fusion, lumbar mobility, patient-reported outcomes, spinal deformity, motion preservation

## Abstract

**Background**: Posterior spinal fusion (PSF) provides reliable deformity correction for adolescent idiopathic scoliosis (AIS); however, it reduces lumbar mobility because of the fusion of motion segments. Hybrid fixation, which combines thoracic PSF with thoracolumbar/lumbar vertebral body tethering (VBT), aims to preserve lumbar function while maintaining satisfactory deformity correction. **Methods**: This retrospective, non-randomized comparative cohort study included 66 adolescents with double-major AIS treated with hybrid fixation (*n* = 30) or posterior long fusion (*n* = 36) at a single tertiary referral spine deformity center. Radiographic outcomes, lumbar flexibility, sagittal range of motion (ROM), lumbar core muscle strength (LCMS), SRS-22r Function scores, complications, and revision surgery were assessed after a minimum follow-up of 24 months. Multivariable regression analyses were used to identify independent predictors of postoperative functional outcomes. **Results**: Both procedures achieved excellent correction of the main thoracic curve and satisfactory global spinal alignment. Posterior long fusion provided greater thoracolumbar/lumbar curve correction (*p* < 0.001), whereas hybrid fixation resulted in significantly better lumbar flexibility, sagittal ROM, and LCMS (all *p* ≤ 0.006). Radiographic tether breakage occurred in eight hybrid patients (26.7%), though most were asymptomatic; revision surgery was required in two patients (6.7% vs. 0%, *p* = 0.20). **Conclusions**: Hybrid fixation was associated with better objective lumbar function than posterior long fusion, with satisfactory deformity correction, in adolescents with double-major AIS. This benefit should be weighed against a higher complication rate driven mainly by tether breakage, supporting individualized, shared decision-making for motion-preserving strategies.

## 1. Introduction

Adolescent idiopathic scoliosis (AIS) is the most common spinal deformity that requires surgical correction in children and adolescents. Posterior spinal fusion (PSF) remains the gold standard surgical treatment because it provides reliable three-dimensional deformity correction and durable long-term outcomes. However, extending fusion into the lumbar spine inevitably sacrifices mobile motion segments, reducing lumbar flexibility and potentially affecting long-term spinal function. Previous studies have demonstrated that more distal fusion levels are associated with greater postoperative loss of lumbar motion, underscoring the importance of preserving lumbar mobility whenever feasible [1,2,3].

Vertebral body tethering (VBT) emerged as a growth-modulating option to fusion, providing a method to straighten the curve while preserving spinal mobility [2,4]. Comparative data indicate that VBT preserves more lumbar mobility, flexibility, and trunk muscle performance than PSF, and patients typically return to physical activity sooner [2,4,5,6]. Although this treatment allows for motion preservation, it comes with certain drawbacks: the correction is often less reliable, and there is a higher incidence of tether-related issues and subsequent surgeries compared to fusion [2,5,6]. A systematic review and meta-analysis, which combined data from 10 comparative studies involving 1168 patients, verified this trade-off. It demonstrated that while VBT offers better motion preservation, it also has a notably higher revision rate compared to PSF [5].

Hybrid fixation, which combines thoracic PSF with lumbar or thoracolumbar VBT, has recently been introduced as a motion-preserving strategy for patients with double-major AIS. The rationale is to combine the reliable correction achieved with thoracic fusion while preserving lumbar motion through anterior tethering. Early clinical series have demonstrated encouraging radiographic correction and acceptable short-term safety, suggesting that hybrid constructs may represent a balanced alternative to conventional PSF and bilateral VBT. Nevertheless, current evidence remains limited to small retrospective cohorts with relatively short follow-up. Furthermore, most published studies have focused primarily on radiographic correction and complication profiles, whereas objective assessments of lumbar mobility, trunk muscle performance, functional capacity, and patient-reported outcomes remain scarce [7].

Therefore, the present study compared radiographic correction, lumbar flexibility, trunk muscle performance, patient-reported outcomes, and complications between hybrid fixation and posterior long fusion in adolescents with double-major AIS. We hypothesized that hybrid fixation would better preserve lumbar function while maintaining satisfactory deformity correction, whereas posterior long fusion would provide greater coronal correction with a lower risk of revision surgery.

## 2. Materials and Methods

### 2.1. Study Design, Setting and Patient Selection

**Figure 1:**
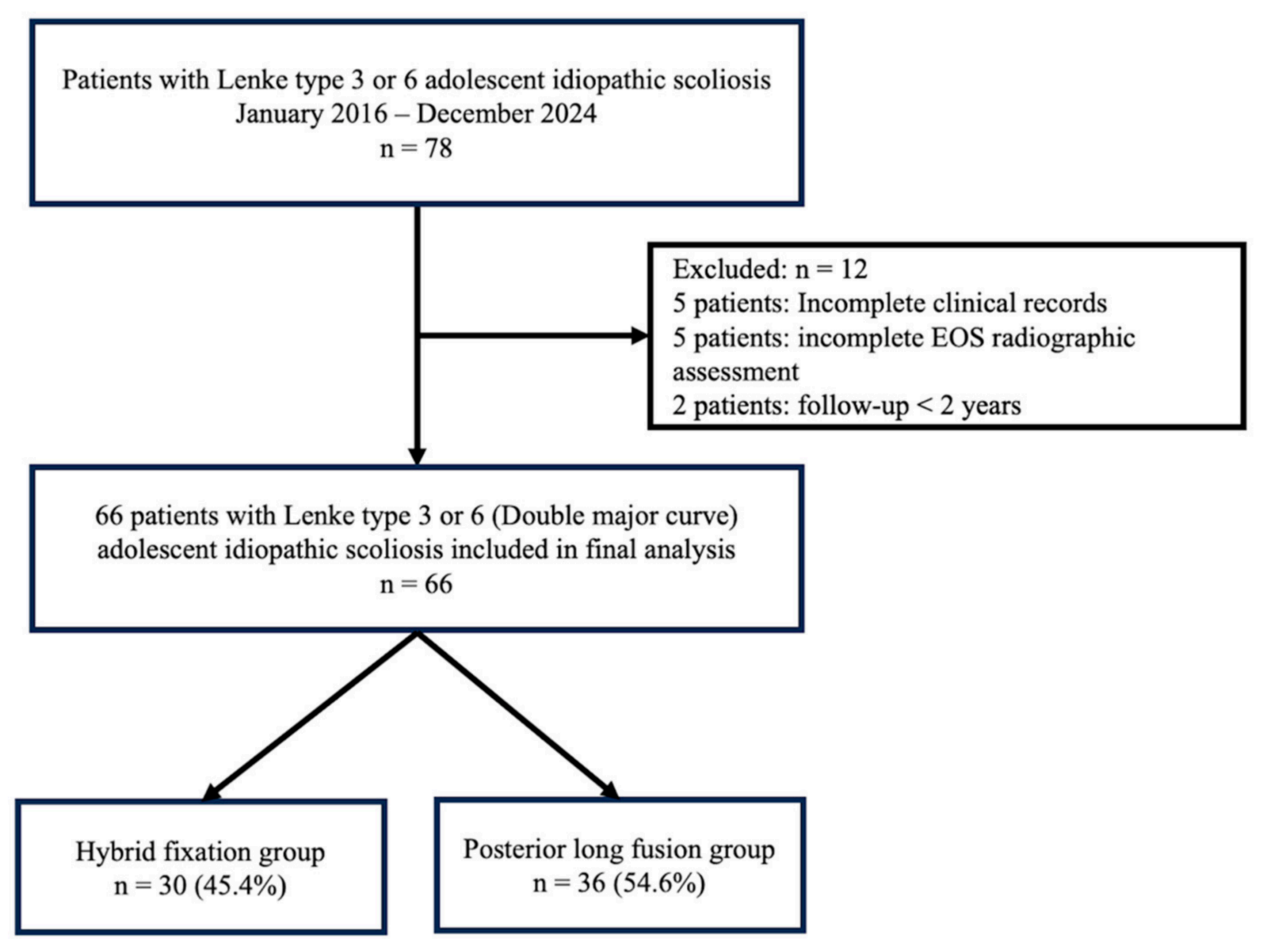
Patient Flow Chart.

This retrospective, non-randomized comparative cohort study was conducted at a single tertiary referral spine deformity center. The institutional spine database was screened for all surgically treated AIS patients between January 2016 and December 2024, and those with Lenke type 3 or 6 double-major curves were identified for eligibility assessment. Patient selection is summarized in Figure 1. Seventy-eight patients met the initial eligibility criteria. After exclusion of 12 patients because of incomplete clinical records (*n* = 5), incomplete EOS (EOS Imaging, Paris, France) radiographic assessment (*n* = 5), or follow-up shorter than 2 years (*n* = 2), 66 patients were included in the final analysis.

This study is reported in accordance with the Strengthening the Reporting of Observational Studies in Epidemiology (STROBE) guidelines for cohort studies. Eligible patients were 10–18 years of age, had Lenke type 3 or 6 double-major AIS, Risser grade 0–4, and a minimum clinical and radiographic follow-up of 24 months. Treatment allocation was based on curve characteristics, skeletal maturity, surgeon recommendation, and shared decision-making involving the treating surgeons, patients, and their families. Patients with previous spinal surgery, congenital, neuromuscular, or syndromic scoliosis, or incomplete follow-up or EOS radiographic assessment were excluded.

All patients followed the same standardized postoperative rehabilitation protocol, including early mobilization, supervised physiotherapy, progressive return to activities of daily living, and return to unrestricted sports after radiographic confirmation of spinal stability according to institutional practice.

Retrospective ethics approval was obtained from the Institutional Review Board (Approval No. 2025-08-03/Date: 30 October 2025). Written informed consent for participation and for publication of anonymized clinical and imaging data was obtained from all participants and their parents or legal guardians prior to data collection. The study was conducted in accordance with the principles of the Declaration of Helsinki.

### 2.2. Surgical Technique

All procedures were performed by the senior author (A.H.) using standardized surgical techniques. Posterior instrumentation in both groups consisted of an all-pedicle screw construct (Medtronic, Minneapolis, MN, USA) with 5.5-mm cobalt–chromium rods.

In the hybrid fixation group, thoracic deformity correction with posterior spinal fusion was followed by thoracoscopic anterior vertebral body tethering using REFLECT™ Scoliosis Correction System (Globus Medical, Audubon, PA, USA) of the thoracolumbar or lumbar curve. The tether was sequentially tensioned from proximal to distal under fluoroscopic guidance to achieve gradual correction while avoiding overcorrection. Tensioning was individualized according to curve magnitude, flexibility, skeletal maturity, and the desired intraoperative correction while preserving the potential for continued growth modulation.

Patients in the posterior long fusion group underwent conventional posterior deformity correction. Fusion levels and the upper and lower instrumented vertebrae were selected according to established deformity correction principles, considering the stable vertebra, coronal balance, sagittal alignment, and lumbar curve characteristics while preserving distal mobile segments whenever feasible. All procedures were performed with continuous multimodal intraoperative neuromonitoring.

### 2.3. Radiographic Evaluation

Standing full-length biplanar EOS radiographs were obtained with patients in a standardized upright position preoperatively and at the standardized 24-month follow-up. Coronal parameters included the main thoracic (MT) Cobb angle, thoracolumbar/lumbar (TL/L) Cobb angle, and coronal balance measured as the horizontal distance between the C7 plumb line and the central sacral vertical line (CSVL). Sagittal parameters included thoracic kyphosis (T5–T12) and lumbar lordosis (L1–S1). Curve correction was calculated as the difference between preoperative and postoperative measurements.

Radiographic measurements were independently performed using the institutional PACS software (Sectra IDS7 PACS (version 26.2.12.8350; Sectra AB, Linköping, Sweden)) by two fellowship-trained spine surgeons blinded to treatment allocation. Interobserver and intraobserver reliability were assessed using the intraclass correlation coefficient (ICC) and demonstrated excellent agreement (interobserver ICC = 0.94, 95% CI 0.90–0.97; intraobserver ICC = 0.96, 95% CI 0.93–0.98). Curve flexibility was assessed on supine side-bending radiographs obtained preoperatively; bending correction was calculated as the percentage reduction in Cobb angle between the standing preoperative and the corresponding side-bending X-ray.

### 2.4. Functional Assessment

All functional assessments, including measurements of lumbar flexibility, lumbar range of motion, and core muscle strength, were conducted by the same physiotherapist (S.A.) at the standardized 24-month follow-up to ensure measurement consistency.

Lumbar flexibility was assessed using the standard Schober test. With the participant standing, the L5 spinous process was identified and marked as the reference point. A second mark was placed 10 cm superior to the first. The participant was then instructed to perform maximal forward trunk flexion while keeping the knees extended. The increase in distance from the initial 10 cm represented lumbar flexion mobility, recorded in centimeters. Lumbar range of motion (ROM) was measured using a universal goniometer. Lumbar flexion was measured in standing, with the pivot positioned at the lateral aspect of the lumbosacral junction, the stationary arm perpendicular to the floor, and the movable arm aligned with the lateral midline of the trunk toward the axilla; hip motion was minimized during the assessment. Lumbar lateral flexion was assessed in standing with the pivot at the midpoint of the lumbosacral junction, the stationary arm aligned parallel to the floor through the posterior superior iliac spine, and the movable arm directed toward the C7 spinous process; trunk compensation was avoided throughout. Lumbar rotation was measured seated (hips and knees at 90°) to minimize pelvic compensation, with the pivot placed over the cranial aspect of the head, the stationary arm aligned parallel to the iliac crests, and the movable arm aligned with the acromion processes. Each measurement was taken three times and averaged. The primary outcome, defined a priori, was total sagittal lumbar ROM (flexion + extension). Intra-rater reliability was confirmed in a preliminary sample (ICC = 0.91, 95% CI 0.74–0.97).

Lumbar core muscle strength (LCMS) was assessed using the Centaur 3D Trunk Muscle Testing System (BfMC GmbH, Leipzig, Germany), a computer-assisted three-dimensional spatial rotation device. The device evaluates the ability of the spinal stabilizing musculature to maintain an upright posture under multidirectional loading conditions by quantifying the momentum generated during controlled tilting movements. Prior to testing, the participant’s pelvis and thighs were stabilized; during the assessment, participants crossed their arms over the chest while holding the control button. Participants were instructed to terminate the test when they experienced pain, excessive strain, or an inability to maintain an upright posture. Each participant performed two trials; the first was conducted for familiarization and the results from the second trial were used for analysis. Tilting was performed in eight directions (0°, ±45°, ±90°, ±135°, and 180°; positive values indicate right, negative values indicate left). Momentum values (Nm/kg) were calculated on the basis of tilt angle, trunk length, body weight, and force exerted relative to the trunk’s deviation from the body axis, and were reported for the posterior, anterior, and lateral directions.

Health-related quality of life was assessed using the Scoliosis Research Society-22 revised (SRS-22r) questionnaire. The SRS-22r Function domain was selected a priori as the patient-reported outcome measure for multivariable analyses.

### 2.5. Complications and Reoperations

All perioperative complications and reoperations occurring within 24 months after the index procedure were recorded. Radiographic tether breakage and overcorrection were predefined and assessed at routine follow-up visits. Reoperation was defined as any unplanned secondary surgical procedure related to the index spinal construct.

### 2.6. Statistical Analysis

Depending on their distribution, continuous variables are reported as mean ± standard deviation or median with interquartile range; categorical variables are given as counts and percentages. Normality was checked with the Shapiro–Wilk test. Group comparisons relied on the independent-samples *t* test or Mann–Whitney U test for continuous data, and on the chi-square or Fisher’s exact test for categorical data, chosen according to the underlying distribution and cell counts.

All consecutive eligible patients meeting the inclusion criteria during the study period were included in the analysis. No imputation was performed for missing data; patients with incomplete datasets were excluded from the final analysis. No a priori sample size calculation was performed because the cohort size was fixed by the number of eligible patients treated during the study period. Given the fixed sample size of 30 and 36 patients, the study had 80% power at a two-sided α of 0.05 to detect a minimum standardized between-group difference of d = 0.70.

Secondary outcomes were considered exploratory, and multiplicity was controlled using the Holm procedure. Multivariable linear regression models were constructed to identify independent predictors of postoperative functional outcomes, including the SRS-22r Function domain. Variables entered into the models were selected a priori based on clinical relevance and included age at surgery, sex, body mass index, baseline MT Cobb angle, baseline TL/L Cobb angle, and Risser grade. Two-sided *p* values < 0.05 were considered statistically significant. Statistical analyses were performed using R version 4.5.1 (R Foundation for Statistical Computing, Vienna, Austria).

## 3. Results

### 3.1. Study Cohort

A total of 66 patients with adolescent idiopathic scoliosis (AIS) and double-major curves met the eligibility criteria and completed the standardized 24-month follow-up assessment. Thirty patients underwent hybrid fixation consisting of thoracic posterior spinal fusion (PSF) combined with thoracolumbar/lumbar vertebral body tethering (VBT), whereas 36 underwent conventional posterior long fusion. Baseline demographic characteristics, curve morphology, skeletal maturity, and preoperative deformity severity were comparable between groups (Table 1). Operative characteristics differed as expected according to construct design: the hybrid group had fewer thoracic fused levels and demonstrated significantly shorter operative time, lower estimated blood loss, and shorter hospital stay than the posterior long fusion group. Follow-up duration was comparable between the groups (30.7 ± 4.6 vs. 32.5 ± 6.1 months, *p* = 0.19) (Table 1). Treatment era distribution was comparable between groups (2016–2019: 10 [33.3%] hybrid vs. 16 [44.4%] fusion; 2020–2024: 20 [66.7%] vs. 20 [55.6%]; *p* = 0.36). Preoperative curve flexibility did not differ significantly (MT bending correction: 44.8 ± 12.3% vs. 42.1 ± 11.7%, *p* = 0.38; TL/L bending correction: 51.5 ± 14.2% vs. 48.0 ± 13.5%, *p* = 0.31). A sensitivity analysis restricted to patients with Risser grade ≥ 2 (*n* = 48) confirmed the primary finding (total sagittal lumbar ROM: 136.5 ± 9.0° vs. 117.8 ± 9.5°, *p* < 0.001).

### 3.2. Radiographic Outcomes

**Figure 2:**
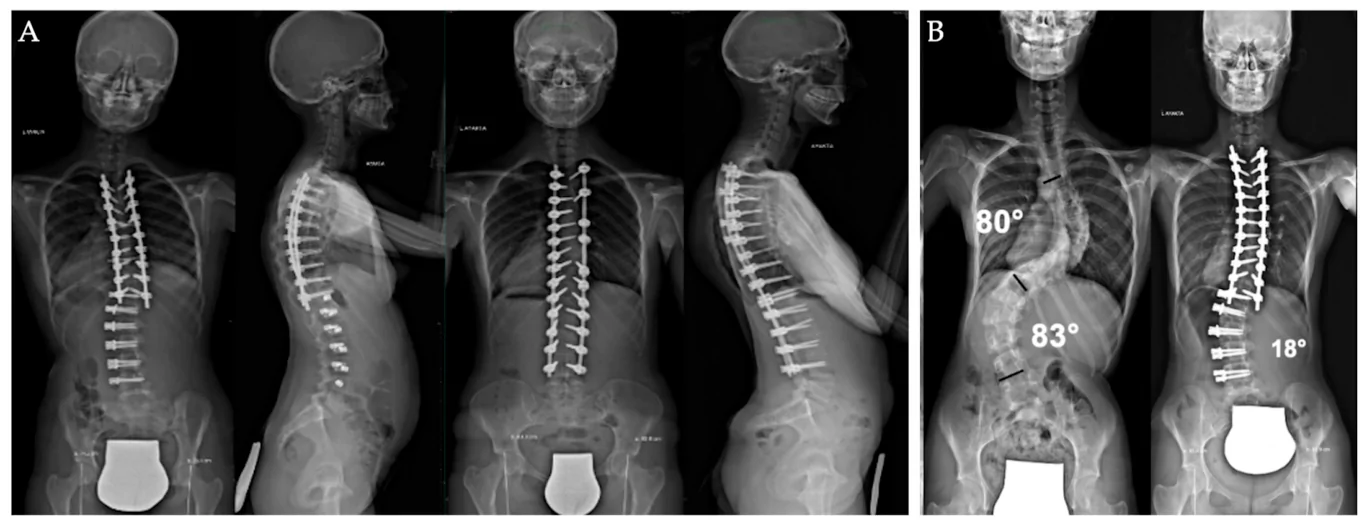
Representative radiographic outcomes following hybrid fixation and posterior spinal fusion. (**A**) Standing whole-spine AP and lateral EOS radiographs of representative patients from the hybrid fixation (**left**) and posterior long fusion (**right**) groups. (**B**) Preoperative (**left**) and 24-month postoperative (**right**) standing AP EOS radiographs of a hybrid fixation patient, demonstrating thoracic posterior spinal fusion combined with thoracolumbar/lumbar vertebral body tethering.

Both surgical strategies achieved substantial correction of the main thoracic deformity and improved coronal balance at 24 months (Table 2). The magnitude of main thoracic curve correction and changes in coronal balance were comparable between groups. Likewise, postoperative thoracic kyphosis and lumbar lordosis did not differ significantly. In contrast, posterior long fusion achieved significantly greater thoracolumbar/lumbar curve correction than hybrid fixation (*p* < 0.001), representing the principal radiographic difference between the two treatment strategies. Representative preoperative and final EOS radiographs are shown in Figure 2.

### 3.3. Functional Outcomes

Objective functional outcomes consistently favored hybrid fixation at the 24-month evaluation (Table 3). Lumbar flexibility measured by the Schober test was significantly greater in the hybrid group than in the posterior long fusion group (4.90 ± 1.10 vs. 3.70 ± 1.00 cm, *p* = 0.002). The primary outcome of interest, total sagittal lumbar range of motion (ROM), was also significantly greater following hybrid fixation (138.0 ± 8.4° vs. 118.2 ± 9.2°, *p* < 0.001), corresponding to a large effect size (Cohen’s d = 2.24, 95% CI 1.62–2.86). Similar advantages were observed across all individual ROM components, including lumbar flexion, extension, bilateral lateral bending, and axial rotation (all *p* < 0.001), with large effect sizes throughout (Cohen’s d 1.11–1.55).

**Figure 3:**
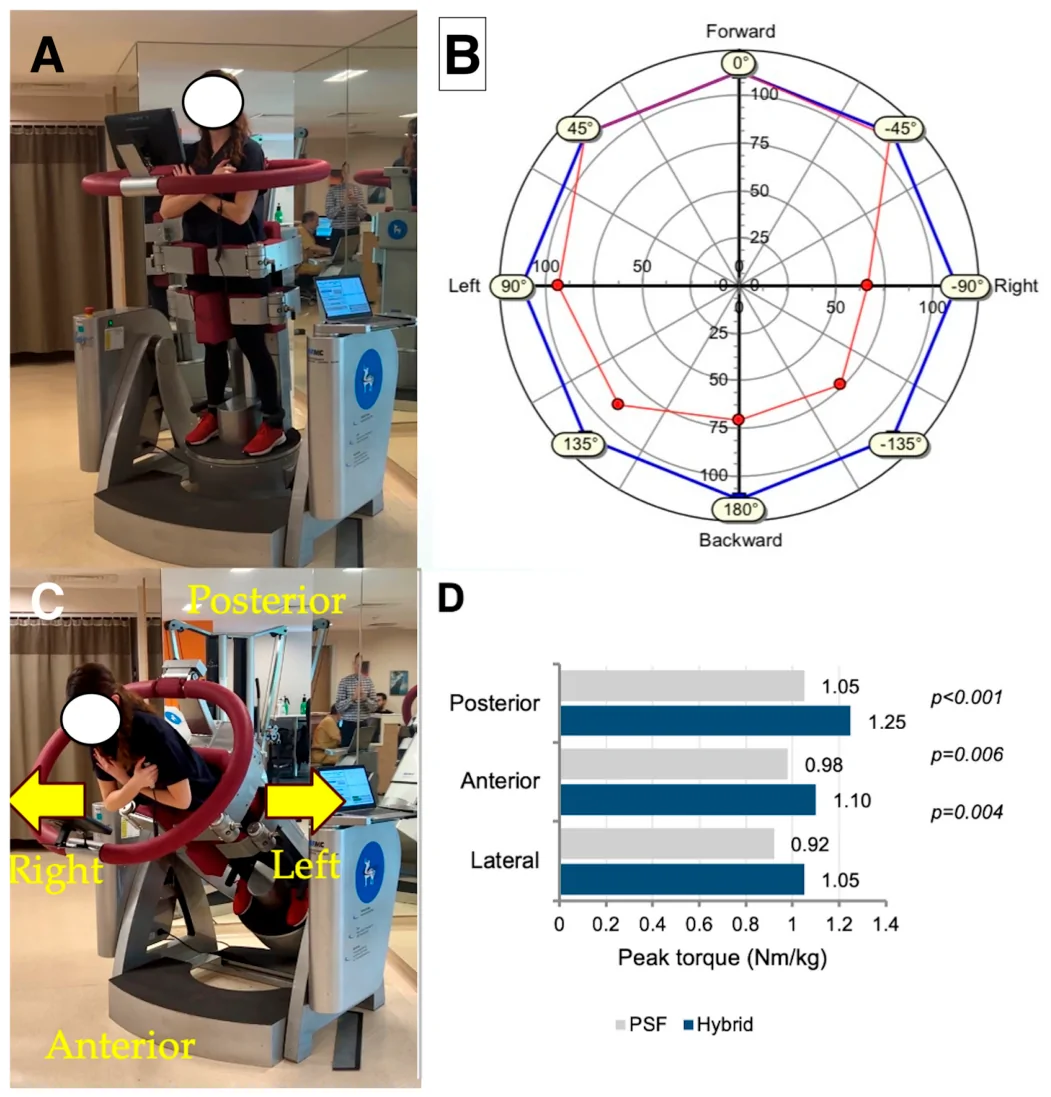
Postoperative assessment of lumbar core muscle strength using the Centaur 3D Trunk Muscle Testing System. (**A**) Representative patient positioning during lumbar core muscle strength assessment. (**B**) Computerized test report generated by the Centaur system; the blue circle represents maximum momentum values and the red circle represents the patient’s measured values. (**C**) Representative image showing the patient’s forward tilt position on the Centaur device (0 degrees). (**D**) Comparison of normalized postoperative lumbar core muscle strength (Momentum, Nm/kg) between the hybrid fixation and posterior spinal fusion groups in the posterior, anterior, and lateral directions.

Hybrid fixation was also associated with superior lumbar core muscle strength. Posterior, anterior, and lateral lumbar core muscle strength measurements were all significantly greater in the hybrid group than in the posterior long fusion group (all *p* ≤ 0.006) (Table 3). Representative Centaur testing and postoperative lumbar core muscle strength are presented in Figure 3. Despite these objective functional advantages, patient-reported function assessed using the SRS-22r Function domain did not differ significantly between groups.

### 3.4. Complications

Overall, 11 patients (36.7%) in the hybrid group experienced at least one complication compared with four (11.1%) in the posterior long fusion group (*p* = 0.02) (Table 4). Perioperative events included one transient neurological deficit and one pleural effusion in the hybrid group, and three superficial wound complications in the fusion group. Radiographic tether breakage occurred in eight hybrid patients (26.7%), of whom six (75%) were asymptomatic and managed with surveillance and two (6.7%) required revision surgery (one re-tethering, one conversion to posterior spinal fusion). One additional patient (3.3%) developed overcorrection, managed with surveillance. No fusion patient required secondary surgery (6.7% vs. 0%, *p* = 0.20) (Table 4).

### 3.5. Multivariable Analysis

After adjustment for the a priori selected covariates, posterior long fusion remained independently associated with inferior functional outcomes at 24 months. Compared with hybrid fixation, posterior long fusion was associated with significantly lower total sagittal lumbar ROM (adjusted β = −16.6, 95% CI −19.7 to −13.5; *p* < 0.001), lower SRS-22r Function domain scores (adjusted β = −0.15, 95% CI −0.28 to −0.02; *p* = 0.024), and lower posterior lumbar core muscle strength (adjusted β = −0.10 Nm/kg, 95% CI −0.19 to −0.01; *p* = 0.031), confirming that the observed functional differences remained significant after multivariable adjustment. In Model 1, Risser grade was also independently associated with total sagittal lumbar ROM (adjusted β = −3.21 per grade, *p* < 0.001). In Model 2, baseline TL/L Cobb angle was the only additional significant predictor of SRS-22r Function (adjusted β = −0.012 per degree, *p* = 0.018). In Model 3, female sex (adjusted β = 0.09, *p* = 0.028) and Risser grade (adjusted β = −0.04, *p* = 0.014) were independently associated with posterior LCMS. Full model details are presented in Table 5.

## 4. Discussion

The main finding was that hybrid fixation was associated with better objective lumbar function than posterior long fusion in patients with double-major AIS. At the 24-month assessment, the hybrid group showed significantly greater lumbar flexibility, total sagittal lumbar range of motion, and core muscle strength, whereas posterior long fusion achieved greater thoracolumbar and lumbar correction and a lower complication rate. Both strategies corrected the main thoracic deformity well and restored global spinal alignment. These findings reflect a trade-off between lumbar motion preservation and long-term construct durability: hybrid fixation is a motion-preserving option for carefully selected patients, not a universal replacement for posterior fusion [7].

Both strategies corrected the main thoracic curve well, but posterior long fusion achieved significantly greater thoracolumbar/lumbar correction. From a biomechanical perspective, it is understandable that rigid pedicle screw constructs offer immediate stability across three columns and exert a greater corrective force than a tether can provide [8,9,10,11]. Earlier comparative studies and systematic reviews have shown a similar pattern: posterior fusion generally achieves a more thorough correction of the coronal curve, whereas tethering compromises some of this correction to maintain motion [8,9,10,12]. Our findings are consistent with these reports: despite less complete lumbar correction, hybrid fixation maintained adequate overall spinal alignment. The substantially greater TL/L coronal correction achieved by posterior long fusion did not translate into significant differences in the measured sagittal-plane parameters; thoracic kyphosis and lumbar lordosis were comparable between groups. Thus, within the sagittal parameters assessed in this study, the residual difference in TL/L coronal correction was not accompanied by a measurable difference in sagittal alignment. A small loss of correction may be acceptable when preserving lumbar mobility is a treatment priority.

Beyond radiographic correction, the most clinically relevant finding was better preservation of objective lumbar function after hybrid fixation. The likely mechanism is straightforward: posterior fusion eliminates motion across instrumented lumbar segments, whereas vertebral body tethering preserves controlled segmental mobility while maintaining spinal alignment [1,4,12,13]. Raitio et al. identified motion preservation as a principal theoretical advantage of vertebral body tethering, though they noted the limited clinical evidence for objective functional outcomes [4]. Wong et al., in a recent systematic review, reported that vertebral body tethering provides less predictable radiographic correction than posterior spinal fusion but consistently better postoperative spinal mobility and function [12]. Our findings extend these observations: the functional advantage persists even when tethering is combined with fusion in a hybrid construct rather than used alone.

Hybrid fixation’s advantage was consistent across every functional assessment—flexion, extension, lateral bending, axial rotation, the Schober test, and trunk muscle strength. This consistency across independent measures strengthens the biological plausibility of the effect and suggests that preserved lumbar motion improves neuromuscular performance as well as flexibility. Two biomechanical studies support this interpretation. Lee et al. showed that extending posterior fusion into the distal lumbar spine progressively reduces postoperative mobility with each additional fused segment [1,14]. Pahys et al. similarly found decreasing lumbar flexibility with more distal fusion levels, underscoring the value of preserving mobile lumbar segments when feasible [14]. Together, these findings support hybrid fixation as a way to maintain lumbar motion while still achieving adequate deformity correction.

Despite the objective functional gains after hybrid fixation, SRS-22r Function scores did not differ significantly between groups. This gap between objective and patient-reported outcomes has been reported before in motion-preserving scoliosis surgery [12,15,16]. One explanation is that the SRS-22r, though well validated for health-related quality of life in AIS, may lack the sensitivity to detect subtle differences in lumbar mobility among otherwise healthy adolescents [17]. Wong et al. reported a similar pattern: improved postoperative spinal mobility after vertebral body tethering was not consistently matched by better patient-reported outcomes [12]. Carreon et al. established small minimum clinically important difference (MCID) thresholds for the SRS Activity domain, and Kelly et al. later showed that the minimum detectable difference exceeded these thresholds—meaning modest functional gains can fall below what the instrument can reliably detect [15,16]. Objective measures of flexibility, range of motion, and muscle strength may therefore be more sensitive than patient-reported questionnaires for evaluating motion-preserving techniques, particularly during early and mid-term follow-up.

These functional advantages must be weighed against a higher composite complication rate. Two patients (6.7%) in the hybrid cohort required revision surgery, compared with none after posterior long fusion. Although this difference did not reach statistical significance, it reflects the known mechanical liabilities of tether-based constructs [18]. Radiographic tether breakage was observed in eight patients (26.7%), consistent with reported rates in the VBT literature, but six of eight (75%) were asymptomatic and managed with surveillance alone [8,19,20,21]. This distinction matters: radiographic tether failure and clinical failure requiring revision are not the same thing, and recent long-term studies confirm that most breakages remain clinically silent [19,20,21]. The composite complication burden nonetheless supports careful preoperative counseling, and patients and families should understand this trade-off before surgery.

These findings have practical implications for patient selection. Posterior long fusion remains preferable when maximum correction and construct durability are the priorities. Hybrid fixation suits adolescents who value lumbar mobility and trunk function more highly, provided they accept a higher likelihood of reoperation—a trade-off that should be made explicit during shared decision-making. Successful use of motion-preserving techniques also depends on appropriate patient selection, guided by skeletal maturity, curve characteristics, flexibility, and patient expectations [12,22,23].

To our knowledge, this is among the few comparative studies of hybrid fixation in double-major AIS that incorporates comprehensive objective functional assessments—including lumbar flexibility, lumbar ROM, Schober test, and trunk muscle strength—alongside radiographic and patient-reported outcomes. The single-team design with standardized surgical and rehabilitation protocols reduces treatment heterogeneity.

The retrospective, non-randomized design carries residual confounding from unmeasured variables such as treatment era, curve flexibility, and family preference; all between-group differences should be interpreted as associations rather than causal effects. Although treatment-era distribution did not differ significantly between groups, temporal changes in surgical experience, patient selection, perioperative management, and rehabilitation may have influenced the observed outcomes; therefore, residual confounding related to treatment era cannot be excluded. The single-center setting limits generalizability, and multicenter replication is needed. The minimum 24-month follow-up may underestimate late tether-related complications. Because the functional assessor was aware of treatment allocation, differential measurement bias between groups cannot be excluded, despite the use of standardized assessment protocols and demonstrated intra-rater reliability (ICC = 0.91). Future prospective comparative studies should therefore incorporate blinded functional outcome assessment whenever feasible. Preoperative functional data were unavailable, precluding within-subject change analysis. Furthermore, the reported lumbar ROM values (138° and 118°) reflect goniometric total lumbar flexion plus extension, which includes contributions from unfused segments and cannot be directly compared with radiographic segmental motion studies. Eyvazov et al. reported similar goniometric lumbar ROM values in unoperated AIS patients, supporting the plausibility of our measurements, although the absence of healthy controls precludes normative comparison [24]. The hybrid construct combines two techniques, so functional benefits cannot be attributed to tethering alone. Finally, although the available sample size was sufficient to detect the prespecified primary functional difference, it remained modest for complication and revision comparisons; therefore, the observed revision difference (6.7% vs. 0%, *p* = 0.20) should be interpreted cautiously. The absence of qualitative patient data and activity-specific patient-reported outcome measures further limits interpretation of the discrepancy between objective functional outcomes and SRS-22r Function scores.

## 5. Conclusions

In conclusion, hybrid fixation was associated with better lumbar flexibility, lumbar range of motion, and trunk muscle strength than posterior long fusion at 24 months, with adequate deformity correction maintained in patients with double-major AIS. Radiographic tether breakage was common (26.7%) but predominantly asymptomatic, and the revision rate (6.7%) was lower than previously reported for isolated VBT, although the composite complication burden warrants discussion during shared decision-making. Hybrid fixation is therefore not a replacement for conventional posterior spinal fusion, but a selective motion-preserving strategy for carefully chosen patients in whom lumbar function is a priority. Future prospective multicenter studies with longer follow-up are needed to confirm the durability of these benefits, refine patient selection, and further define the role of hybrid fixation in managing double-major adolescent idiopathic scoliosis.

## Figures and Tables

**Table 1:** Baseline characteristics and surgical details by group.

Variable	Hybrid (Thoracic PSF + TL/L VBT) (*n* = 30)	Posterior Long Fusion (*n* = 36)	*p* Value *
Demographics			
Age at surgery, years	13.7 ± 1.6	14.1 ± 1.7	0.31
Sex, female, *n* (%)	22 (73.3)	26 (72.2)	0.92
BMI, kg/m^2^	20.2 ± 2.3	20.6 ± 2.5	0.48
Curve characteristics			
Lenke type, *n* (%)			0.64
3	18 (60.0)	21 (58.3)	
6	12 (40.0)	15 (41.7)	
Skeletal maturity			
Risser grade, median (IQR)	2 (1–3)	3 (2–4)	0.07
Menarche (female only), *n* (%)	15/22 (68.2)	20/26 (76.9)	0.49
Surgical details			
Distal instrumented vertebra, *n* (%)			
L3	18 (60.0)	20 (55.6)	0.71
L4	12 (40.0)	16 (44.4)	
Mean number of thoracic fused levels, *n*	8.4 ± 1.2	11.4 ± 1.3	<0.001
TL/L tethered levels, *n*	4.8 ± 1.9	N/A	
Operative time, minutes	275 ± 55	310 ± 60	0.02
Estimated blood loss, mL	480 ± 180	620 ± 210	0.01
Length of stay, days	4.2 ± 1.1	4.8 ± 1.2	0.04
Follow-up, months	30.7 ± 4.6	32.5 ± 6.1	0.19

Footnotes: * *p* values compare Hybrid versus Posterior long fusion unless otherwise specified. Values are presented as mean ± standard deviation, median with interquartile range, or number and percentage, as appropriate. Menarche status was analyzed among female patients only. *p* values compare hybrid fixation and posterior long fusion. N/A = not applicable.

**Table 2:** Radiographic outcomes preoperative vs. postoperative 24 months by surgical group.

Parameter	Hybrid Preop	Hybrid 24 mo	Hybrid Δ (95% CI)	Fusion Preop	Fusion 24 mo	Fusion Δ (95% CI)	*p* Value †
Coronal plane							
Main thoracic Cobb (°)	48.0 ± 9.0	18.0 ± 7.5	−30.0 (−32.8 to −27.2)	50.5 ± 10.0	16.0 ± 6.5	−34.5 (−37.2 to −31.8)	0.22
Thoracolumbar/Lumbar Cobb (°)	42.0 ± 8.5	22.0 ± 9.0	−20.0 (−22.9 to −17.1)	44.0 ± 9.5	12.0 ± 7.5	−32.0 (−34.6 to −29.4)	<0.001
Coronal balance (C7–CSVL, mm)	14.0 ± 9.0	9.0 ± 7.0	−5.0 (−8.1 to −1.9)	12.0 ± 8.0	7.0 ± 6.0	−5.0 (−7.4 to −2.6)	0.21
Sagittal plane							
Thoracic kyphosis (T5–T12, °)	24.0 ± 8.0	28.0 ± 9.0	+4.0 (+1.1 to +6.9)	23.0 ± 9.0	30.0 ± 9.0	+7.0 (+4.3 to +9.7)	0.37
Lumbar lordosis (L1–S1, °)	50.0 ± 10.0	52.0 ± 10.0	+2.0 (−0.9 to +4.9)	49.0 ± 11.0	51.0 ± 10.0	+2.0 (−0.8 to +4.8)	0.68

Footnotes: † *p* value for between-group comparison of postoperative 24-month values (independent-samples *t* test or Mann–Whitney U test, as appropriate). Δ represents the mean within-group change (postoperative minus preoperative); negative values indicate correction. 95% confidence intervals for Δ were derived from paired *t* tests.

**Table 3:** Functional outcomes at the postoperative 24-month follow-up.

Outcome	Hybrid (*n* = 30)	Fusion (*n* = 36)	*p* Value †	Holm *p*	Cohen’s d (95% CI)
Lumbar flexibility					
Schober test (cm)	4.90 ± 1.10	3.70 ± 1.00	0.002	0.008	1.15 (0.62–1.67)
Lumbar ROM, in °					
Flexion, °	111.6 ± 12.0	97.0 ± 11.5	<0.001	<0.001	1.24 (0.72–1.77)
Extension, °	26.4 ± 5.2	21.2 ± 4.2	<0.001	<0.001	1.11 (0.59–1.63)
Total sagittal Lumbar ROM, ° *	138.0 ± 8.4	118.2 ± 9.2	<0.001	—	2.24 (1.62–2.86)
Side bending, left, °	30.8 ± 6.0	21.9 ± 5.5	<0.001	<0.001	1.55 (1.00–2.10)
Side bending, right, °	30.0 ± 6.1	21.1 ± 5.6	<0.001	<0.001	1.53 (0.98–2.08)
Rotation, left, °	44.9 ± 7.5	35.1 ± 7.0	<0.001	<0.001	1.36 (0.82–1.89)
Rotation, right, °	47.1 ± 7.8	37.3 ± 7.2	<0.001	<0.001	1.31 (0.78–1.84)
LMCS (Nm/kg)					
Posterior LCMS, Nm/kg	1.25 ± 0.20	1.05 ± 0.18	<0.001	<0.001	1.06 (0.54–1.57)
Anterior LCMS, Nm/kg	1.10 ± 0.18	0.98 ± 0.16	0.006	0.012	0.71 (0.21–1.21)
Lateral LCMS, Nm/kg	1.05 ± 0.17	0.92 ± 0.15	0.004	0.012	0.82 (0.31–1.32)
Patient-reported function					
SRS 22r Function domain	4.3 ± 0.4	4.1 ± 0.7	0.112	0.112	0.34 (−0.15–0.83)

Footnotes: † Primary endpoint was total sagittal lumbar ROM (Flexion + Extension) at postoperative 24 months. * Primary outcome; tested at uncorrected α = 0.05 and excluded from the Holm correction family. All remaining outcomes were considered secondary and exploratory; the Holm procedure was applied across this family of 11 comparisons to control the family-wise error rate, and Holm-adjusted *p* values are reported alongside raw *p* values. — = not applicable. Raw *p* values were derived from independent-samples *t* test or Mann–Whitney U test, as appropriate. Lumbar core muscle strength was assessed using the Centaur 3D Trunk Muscle Testing System, which quantifies momentum generated during controlled multidirectional tilting. Momentum values were calculated in N·m and normalized to body mass (N·m/kg). Cohen’s d was calculated using the pooled standard deviation; values of 0.2, 0.5, and 0.8 correspond to small, medium, and large effects, respectively. Positive effect sizes favor hybrid fixation.

**Table 4:** Complications and reinterventions within 24 months.

Event	Hybrid (*n* = 30)	Fusion (*n* = 36)	*p* Value †
Any complication, *n* (%)	11 (36.7)	4 (11.1)	0.020
Perioperative (≤30 days), *n* (%)	2 (6.7)	3 (8.3)	0.792
Transient neurological deficit	1 (3.3)	—	
Pleural effusion	1 (3.3)	—	
Superficial wound complication	—	3 (8.3)	
Radiographic tether breakage, *n* (%)	8 (26.7)	—	—
Asymptomatic (surveillance)	6 (20.0)	—	
Requiring revision surgery	2 (6.7)	—	
Overcorrection, *n* (%)	1 (3.3)	—	—
Revision surgery, *n* (%)	2 (6.7)	0 (0)	0.203
Conversion to posterior spinal fusion, *n* (%)	1 (3.3)	—	—

Footnote: † *p* values were calculated using the chi-square test, Fisher’s exact test, or Mann–Whitney U test, as appropriate. Fisher’s exact test was used when expected cell counts were <5. All events were captured within 24 months after index surgery. “Any complication” was defined as any patient experiencing at least one adverse event; patients may appear in more than one event category but are counted only once in this composite row. Tether breakage was defined radiographically as a suspected breakage based on an interscrew angle change of at least 5 degrees between adjacent screws compared with the immediate postoperative radiograph, or a visible discontinuity of the tether when present, and was classified as asymptomatic (managed with surveillance) or requiring revision surgery. Overcorrection was defined as a postoperative coronal curve that crossed neutral to the opposite direction or progressed beyond 10 degrees in the opposite direction on standing radiographs, with or without clinical indication for intervention.

**Table 5:** Multivariable linear regression analyses for the selected functional outcomes at 24 months.

Predictor	Adjusted β	95% CI	*p* Value
Model 1: Total sagittal lumbar ROM, ° (*n* = 66; R^2^ = 0.71; Adjusted R^2^ = 0.68)			
Posterior long fusion vs. Hybrid fixation	−16.60	−19.70 to −13.50	<0.001
Age at surgery, years	−1.42	−2.88 to 0.04	0.057
Sex, female vs. male	1.85	−1.94 to 5.64	0.332
Body mass index, kg/m^2^	−0.53	−1.30 to 0.24	0.174
Baseline main thoracic Cobb angle, °	0.06	−0.12 to 0.24	0.498
Baseline TL/L Cobb angle, °	−0.18	−0.38 to 0.02	0.078
Risser grade	−3.21	−5.04 to −1.38	<0.001
Model 2: SRS-22r Function domain (*n* = 66; R^2^ = 0.18; Adjusted R^2^ = 0.08)			
Posterior long fusion vs. Hybrid fixation	−0.15	−0.28 to −0.02	0.024
Age at surgery, years	0.02	−0.04 to 0.08	0.512
Sex, female vs. male	0.08	−0.10 to 0.26	0.367
Body mass index, kg/m^2^	−0.03	−0.07 to 0.01	0.118
Baseline main thoracic Cobb angle, °	0.002	−0.006 to 0.010	0.631
Baseline TL/L Cobb angle, °	−0.012	−0.022 to −0.002	0.018
Risser grade	0.06	−0.03 to 0.15	0.172
Model 3: Posterior LCMS Nm/kg (*n* = 66; R^2^ = 0.52; Adjusted R^2^ = 0.46)			
Posterior long fusion vs. Hybrid fixation	−0.10	−0.19 to −0.01	0.031
Age at surgery, years	−0.02	−0.04 to 0.01	0.128
Sex, female vs. male	0.09	0.01 to 0.17	0.028
Body mass index, kg/m^2^	−0.008	−0.022 to 0.006	0.248
Baseline main thoracic Cobb angle, °	0.001	−0.003 to 0.004	0.724
Baseline TL/L Cobb angle, °	−0.002	−0.006 to 0.002	0.312
Risser grade	−0.04	−0.07 to −0.01	0.014

Footnotes: Adjusted β estimates were derived from multivariable linear regression models. All dependent variables were assessed at the standardized postoperative 24-month visit. The treatment group variable (posterior long fusion vs. hybrid fixation, reference) was entered as the primary predictor. Models were adjusted for age at surgery, sex, body mass index, baseline main thoracic Cobb angle, baseline thoracolumbar or lumbar Cobb angle, and Risser grade. Negative coefficients indicate lower 24-month values in the posterior long fusion group relative to the hybrid fixation group. Lumbar core muscle strength was measured using the Centaur 3D Trunk Muscle Testing System.

## Data Availability

The data presented in this study are available on request from the corresponding author. The data are not publicly available due to ethical and privacy restrictions related to patient confidentiality and institutional approval requirements.
